# Adaptation aftereffects reveal how categorization training changes the encoding of face identity

**DOI:** 10.1167/jov.20.10.18

**Published:** 2020-10-16

**Authors:** Fabian A. Soto, Karla Escobar, Jefferson Salan

**Affiliations:** Florida International University, Department of Psychology, Miami, FL, USA; Virginia Tech, Psychology Department, Blacksburg, VA, USA

**Keywords:** categorization, face identity, face encoding, adaptation

## Abstract

Previous research suggests that learning to categorize faces along a novel dimension changes the perceptual representation of such dimension, increasing its discriminability, its invariance, and the information used to identify faces varying along the dimension. A common interpretation of these results is that categorization training promotes the creation of novel dimensions, rather than simply the enhancement of already existing representations. Here, we trained a group of participants to categorize faces that varied along two morphing dimensions, one of them relevant to the categorization task and the other irrelevant to the task. An untrained group did not receive such categorization training. In three experiments, we used face adaptation aftereffects to explore how categorization training changes the encoding of face identities at the extremes of the category-relevant dimension and whether such training produces encoding of the category-relevant dimension as a preferred direction in face space. The pattern of results suggests that categorization training enhances the already existing norm-based coding of face identity, rather than creating novel category-relevant representations. We formalized this conclusion in a model that explains the most important results in our experiments and serves as a working hypothesis for future work in this area.

## Introduction

**Figure 1.**
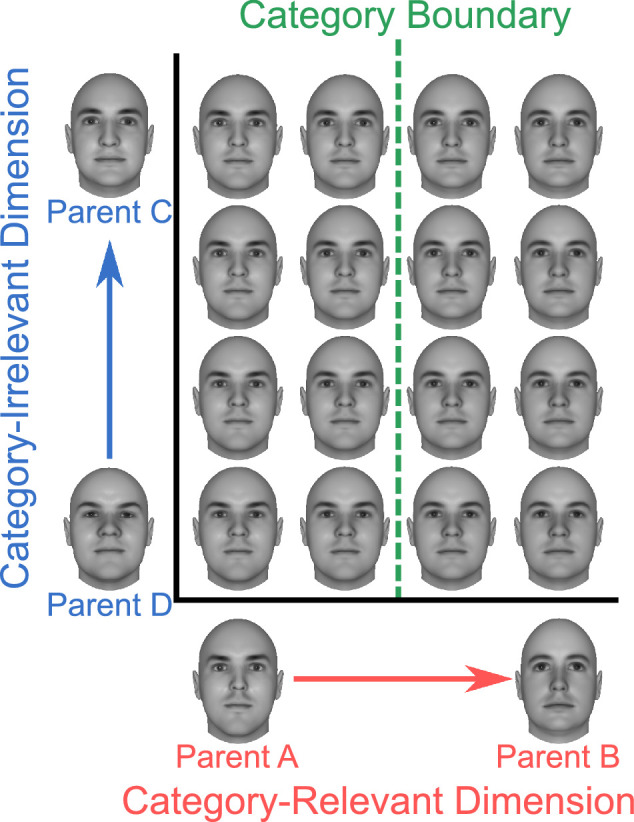
A two-dimensional face morph space. The space is created by first morphing two pairs of parent faces in several steps (Parents A and B; Parents C and D) and then obtaining all possible combinations of the two morphed dimensions. The space can be used to train participants in a categorization task, represented by the green boundary, in which faces to one side of the boundary are assigned to one category and faces on the other side of the boundary are assigned to another category.

Previous research suggests that learning to categorize faces and other objects along a novel dimension changes the perceptual representation of the category-relevant dimension (Goldstone & Steyvers, 2001; Soto & Ashby, 2015; Soto, 2019; see also Folstein et al., 2012, 2014). Such studies have created a novel dimension by morphing two different unfamiliar “parent” faces in several steps to obtain a morphed face dimension. As shown in Figure 1, two of such dimensions can be created, and combinations of several levels of both dimensions result in faces that vary in combinations of four parent faces. Such face dimensions are known to be integral (Soto & Ashby, 2015), meaning that the combined faces are perceived as unique identities rather than solely varying along two dimensions. Faces created using such a morph space can be presented in a categorization task, represented by the green boundary in Figure 1, which divides members of one category (e.g., those more similar to Parent A) from members of a second category (e.g., those more similar to Parent B). In their seminal work, Goldstone and Steyvers (2001) showed that training in such a categorization task produces dimension differentiation, meaning that the category-relevant and category-irrelevant dimensions lose their integrality and instead become represented as special directions in the morphed space (see also Folstein et al., 2012; Soto & Ashby, 2015).

A wealth of evidence demonstrates that dimension differentiation produces changes in the perceptual representation of the stimuli involved (for recent reviews, see Goldstone & Hendrickson, 2010; Goldstone et al., 2008). Stimulus components that are relevant for category discrimination become more distinctive, in what has been termed as “acquired distinctiveness.” Acquired distinctiveness is observed as an increase in discriminability along the category-relevant dimension after categorization training, in perceptual tasks that do not require categorization (Folstein et al., 2012, 2013, 2014; Goldstone & Steyvers, 2001; Van Gulick & Gauthier, 2014). In contrast, stimulus components that are irrelevant for category discrimination become less distinctive, in what has been termed “acquired equivalence.” Acquired equivalence is observed as a decrease in discriminability along the category-irrelevant dimension after categorization training. Relatedly, categorization training increases the separability or invariance of the category-relevant dimension (Soto & Ashby, 2015), which means that changes in an irrelevant dimension do not interfere with perception of the category-relevant dimension, according to a variety of tests from multidimensional signal detection theory (Ashby & Soto, 2015; Soto et al., 2017).

Recently, it has been found that the changes of the perceptual representation of faces varying along a novel identity dimension can be explained by changes in the internal template used for face identification (Soto, 2019). By using reverse correlation to estimate internal templates, this study determined that categorization training altered the internal templates used for face identification despite the fact that identification and categorization tasks impose different demands on the visual system (Schyns et al., 2002). After categorization training, internal templates became more invariant across changes in the irrelevant dimension, which relates to the previously found increase in perceptual separability (Soto & Ashby, 2015). The changes produced to an individual's internal template as a result of category learning further suggests that the representation of facial identity can be modified by categorization training.

A common interpretation of this body of research is that categorization training results in the creation of novel features and dimensions that are useful for performance in the categorization task but become stable representations available for use in other tasks as well (Goldstone & Steyvers, 2001; Folstein et al., 2013, 2015; Schyns et al., 1998). For example, Goldstone and Steyvers (2001) interpreted their results as indicating the existence of a mechanism “by which dimensions that are originally psychologically fused together become separated” (p. 117). They explicitly distinguished such mechanism from attentional weighting of dimensions, indicating that dimension differentiation would precede selective attention for stimuli that are not initially perceived as composed of separable dimensions. This was taken as supporting the view that the features and dimensions used by categorization and object recognition are not fixed but sometimes created *de novo* during learning (Schyns et al., 1998). In more recent studies (Folstein et al., 2012; Soto & Ashby, 2019, 2015), researchers have aligned with this interpretation of dimension differentiation as resulting from the development of a novel dimensional structure in the stimuli. Here, we will call this the *dimension creation* hypothesis. An important piece of evidence in favor of it is that the directions in stimulus space that become perceptually differentiated after categorization can be arbitrarily chosen by the experimenter (Goldstone & Steyvers, 2001; Folstein et al., 2012). Dimension creation would also explain why the effects of categorization training can be measured using orthogonal tasks, both through psychophysics (e.g., Goldstone & Steyvers, 2001; Folstein et al., 2012; Soto & Ashby, 2015; Soto et al., 2017) and neuroimaging (Brants et al., 2016; Folstein et al., 2013), and why, in the latter case, effects are observed in high-level visual cortex. An alternative interpretation, which has received less attention in the literature, is that categorization training produces alterations in already existing visual representations, simply enhancing the prelearning selectivity of populations of neurons that happened to provide useful information for the categorization task (Brants et al., 2016). Here, we will call this the *dimension enhancement* hypothesis. From this point of view, the known integrality of morphed dimensions (Goldstone & Steyvers, 2001; Blunden et al., 2015; Soto & Ashby, 2015) would be only apparent, perhaps produced by the fact that stimuli such as faces can be described in a very high-dimensional space, with only a few dimensions aligning with the structure of a categorization task.

**Figure 2.**
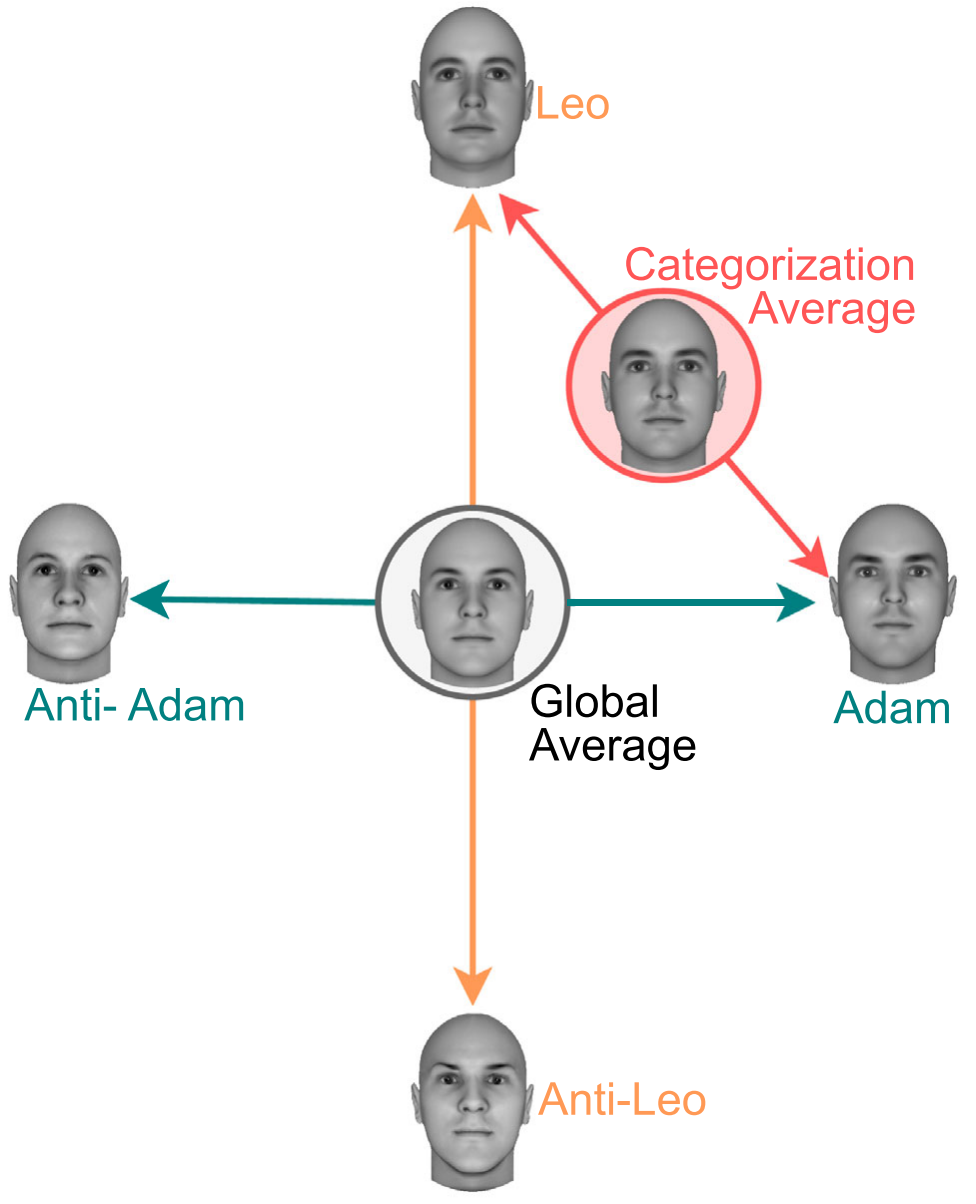
Representation of a number of face identities in face space. The global average face serves as a point of reference for encoding of all other identities. In norm-based coding, face information is encoded by an opponent system, with two neural populations or channels tuned to opposite extremes from the prototype. Thus, if there is a population of neurons tuned to “Adam,” then there is an equivalent but opposite population of neurons tuned to “anti-Adam.” Morphing between two parent faces, such as Adam and Leo, is used to create a morphed face dimension (red line), and an important open question is whether training in a categorization task in which that dimension differentiates the two categories produces a new code, sensitive to changes along that specific direction of face space.

The most accepted theory of how faces are encoded by the visual system proposes that a face is represented as a point in a multidimensional space, or face-space, with distances between faces representing their perceptual discriminability (for a recent review, see Valentine et al., 2016). Encoding of face identity within this space has been proposed to be norm-based, a code in which individual faces are represented by how they deviate from an average or prototype face (Webster & MacLeod, 2011). Figure 2 shows a sketch of how faces would be represented in face space. Here, the global average face serves as the origin of the space, with identities such as “Adam” and “Leo” being represented with respect to that origin. While the specifics of how face norms are encoded are still debated (e.g., Ross et al., 2013), there is consensus among researchers that there is a special role for the average (encoded implicitly or explicitly) in face encoding. Norm-based coding can be implemented by an opponent system in which information about a particular identity is represented by two neural populations or channels tuned to opposite extremes from the prototype. Thus, if there is a population of neurons tuned to “Adam” in Figure 2, then there is an equivalent but opposite population of neurons tuned to “anti-Adam.”

One technique that is often used to study face encoding using psychophysics is face adaptation. Adaptation temporarily alters the sensitivity of the visual system to a stimulus feature in response to exposure to it, generating perceptual aftereffects (Rhodes, 2017; Webster, 2015). Adaptation is commonly used as a tool to directly measure the way faces are encoded by the visual system, as it is considered a way to desensitize the contribution of a specific visual neural population to a discrimination task. This is a reasonable assumption, although the neural mechanisms of adaptation go beyond simple response suppression (Kohn, 2007).

Norm-based encoding is supported by studies showing that recognition of a face is facilitated by adaptation to its anti-face (Leopold et al., 2001). Adapting to an anti-face reduces sensitivity to that face, causing a brief bias in perception away from that identity and toward its corresponding face. Also consistent with opponent-channel coding, the more extreme the anti-face, the larger the perceptual bias that is observed (Jeffery et al., 2018; McKone et al., 2014). In addition, adaptation to a face produces a stronger aftereffect along the morphing dimension going to its corresponding anti-face (through the average) than along a dimension going to a second face, even when dissimilarity is matched (Rhodes & Jeffery, 2006). Finally, evidence for such a code has been found in the human fusiform face area (Loffler et al., 2005; Carlin & Kriegeskorte, 2017) as well as in monkey inferior temporal cortex (Leopold et al., 2006; Freiwald et al., 2009). In line with such findings, it is commonly assumed that studies of face encoding target representations stored in face-selective areas within inferior temporal cortex, at the latest stages of processing in the visual ventral stream (Jiang et al., 2006; Giese & Leopold, 2005).

If the *dimension creation* hypothesis is correct, then this should have important consequences for the encoding of face identity. In particular, categorization training should create a preference for encoding a direction in space connecting the two parents of the category-relevant dimension. For example, if the parents of the category-relevant dimension are Adam and Leo from Figure 2, then a consequence of dimension creation is that the direction in face space connecting these two faces, shown in red in the figure, should be preferentially represented after categorization training. Because the categorization bound is usually placed right in the middle of the category-relevant dimension, the stimulus labeled “categorization average” in Figure 2, which corresponds to that bound, might become an important reference point against which surrounding stimuli are compared. This new encoding of the parent faces of the category-relevant dimension would be at odds with their usual norm-based encoding, and thus it might interfere with it. That is, while the usual encoding of a parent face such as Adam from Figure 2 is in relation to the global average, and opposed to the encoding of anti-Adam, if a novel category-relevant dimension is created, then the encoding of Adam is in relation to the categorization average and opposed to the encoding of Leo, the second parent face. This would be similar to the case of natural face categories such as gender, ethnicity, and expression, which seem to be encoded through their own category-specific norms (Benton et al., 2007; Hsu & Young, 2004; Rutherford et al., 2008; Webster et al., 2004), which are independent from the identity norm.

On the other hand, the *dimension enhancement* hypothesis would predict that the changes produced by categorization training would simply modify the already-existing representation of the parent faces, without creating any novel preferred dimensions or reference points in face space.

The goal of the present study was to explore how categorization training changes the encoding of face identities at the extremes of the category-relevant dimension, as measured through face adaptation aftereffects. Our experiments provide relevant information to start answering the following question: Are the perceptual changes observed after categorization training due to new dimension creation or preexisting dimension enhancement? In Experiment 1, we used a paradigm developed by Leopold et al. (2001) to test whether categorization training changes the norm-based coding of face identity. Experiment 2a was designed to test the same question more thoroughly, while additionally testing whether categorization training produces a preference to encode the category-relevant dimension in face space, with the categorization bound as a reference point. Finally, in Experiment 2b, we used cross-adaptation (i.e., effect of changes across stimuli in an irrelevant dimension on the adaptation effect that they induce along a target dimension; see Ellamil et al., 2008; Fox & Barton, 2007; Fox et al., 2008; Webster, 2015) to test whether categorization training made the encoding of identity more invariant to changes in information about irrelevant faces, as suggested by prior work (Soto & Ashby, 2015; Soto, 2019).

## Experiment 1

**Figure 3.**
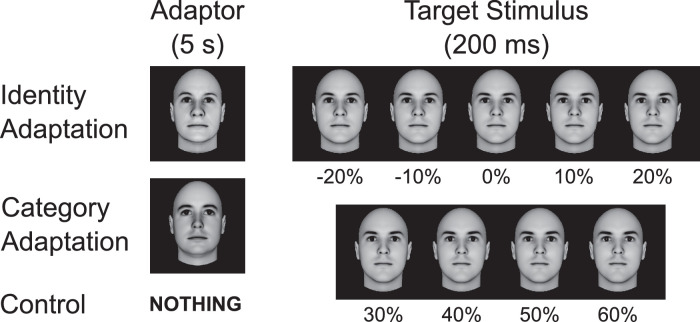
Design of the adaptation test in Experiment 1. Participants were asked to determine whether the briefly presented target stimulus was a particular identity (“Adam” from Figure 2) or not. That identity was at one extreme of the category-relevant dimension in our categorization task. In different blocks, participants were presented with no adaptor in the control condition, with anti-Adam in the Identity Adaptation condition, or with the face at the other extreme of the category-relevant dimension (“Leo” from Figure 2) in the Category Adaptation condition. Targets varied along the direction in face space connecting Adam and anti-Adam through the average face (see Figure 2), in steps of 10%.

Leopold et al. (2001) were the first to offer evidence of norm-based encoding of face identity, using an adaptation design similar to that shown in Figure 3. They trained people to identify a target face, such as the face labeled “Adam” in the figure, and then showed a number of morphed faces along the dimension linking that target face to the face average (i.e., the teal line in Figure 2). The proportion of “Adam” identifications as a function of morph level produces a psychometric function, which can be used to determine identification thresholds. Leopold et al. obtained such psychometric functions in two conditions depicted in Figure 3. In a control condition, they presented only the target stimuli very briefly, whereas in an identity adaptation condition, they first presented an anti-face as an adaptor for 5 s before the presentation of the target. In our example, this adaptor would correspond to “anti-Adam” from Figure 2.

In our first experiment, we used a variation of this design to test whether categorization training modifies the norm-based encoding of face identity. Our target stimulus was one of the parents of the category-relevant dimension shown in Figure 1 (Parent A, or “Adam” from Figure 2). As shown in Figure 3, we included the control and identity adaptation conditions from Leopold et al. (2001). In addition, we included a condition in which the second parent of the category-relevant dimension (“Leo” from Figure 2) was used as an adaptor (category adaptation in Figure 3). We presented a range of target stimuli along the dimension going from the average to the target face, which allowed us to obtain psychometric functions from each participant in all three conditions.

Recently, it has been highlighted that adaptation can influence decision-making processes rather than, or in addition to, sensory processes (Storrs, 2015; Storrs & Arnold, 2012; Witthoft et al., 2018). For this reason, we fit the data to a signal detection model of the psychometric function, which allows us to dissociate sensitivity and decisional contributions to the shape of psychometric curves. Our goal with this was to make sure that our procedures targeted perceptual rather than decisional effects.

**Figure 4.**
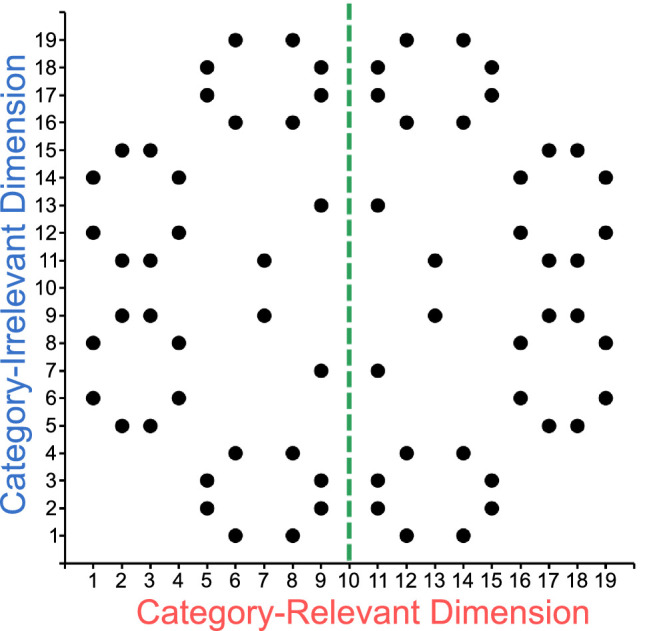
Representation of the categorization task in the two-dimensional morphing space. Points represent stimuli obtained from a specific combination of levels of each dimension. The green dotted line represents the category boundary used for training, so that stimuli on each side of the boundary were assigned to a different category.

There were two groups of participants. The trained group was exposed to extended training with the categorization task shown in Figure 4, whereas the untrained group did not have such exposure. Comparison between groups allowed us to answer two questions. First, whether categorization training reduces norm-based encoding of the target face, as it would be evident from a smaller identity adaptation effect in the trained than in the untrained group. Second, whether categorization training increases a link between the representations of the two parents of the category-relevant dimension, so that adaptation to one would produce an adaptation effect on the other. Such a link has been found with natural categories, like emotion and sex (e.g., Hsu & Young, 2004; Pond et al., 2013; Rutherford et al., 2008; Webster et al., 2004), and it would be evident by a larger category adaptation effect in the trained than in the untrained group.

### Methods

#### Participants

Thirty-eight students at Florida International University (66% female; ages between 18 and 29 years old, median = 23) participated in this experiment in exchange for a monetary compensation (U.S. $10/hour). Half of the participants were semi-randomly assigned to the trained or untrained groups. Nineteen participants (10 in the trained group, 9 in the untrained group) had previously participated in a related study testing the effect of categorization training on face identity representation (Soto, 2019). This prior study finished 2 months before the current study started, but the exact time lapsed between experiments was different for different participants. The previous study used the same stimuli as the current studies in the categorization task (see below), but testing stimuli and procedures were different. All participants previously trained in the categorization task were placed in the trained group and retrained for the current study. Thirteen additional participants took part in the experiment but did not finish it either because they could not achieve good task performance after several sessions or due to attrition. Performance criteria to be included in the last part of the experiment (adaptation testing) were 65% correct in the last session of categorization training and 85% correct in the identification training session (see below).

#### Stimuli

Stimuli were created from images chosen from a database of 300 computer-generated Caucasian faces described by Oosterhof and Todorov (2008), created using the Facegen Modeller program (https://facegen.com/) v. 3.1. Thirty male faces that had similar eyebrow color and levels of facial fat were chosen from the original database. From these 30 faces, three sets of stimuli were created, each to be used in a different experimental task.

The first stimulus set was created to be used in the categorization training task. Judgments of similarity between all faces were obtained in a pilot study, described in detail by Soto (2019). Two pairs of faces from the stimulus set were selected to be used as parents in the subsequent construction of the morphed multidimensional stimuli, so that the mean dissimilarity of the first pair (0.51, *SD* = 0.13) was close to the mean dissimilarity of the second pair (0.49, *SD* = 0.13). This ensured that the pairs of parent faces would create dimensions of relatively similar salience. The selected faces were also not easily discriminable by any salient feature, such as degree of femininity/masculinity or head width. All faces were converted to grayscale and their intensity histograms were equalized to ensure that the stimuli along the resulting morphed dimensions varied in shape features but not in low-level features such as color or brightness.

A two-dimensional space was created using a factorial morphing procedure (Folstein et al., 2012; Goldstone & Steyvers, 2001; Soto & Ashby, 2015) to generate morphed faces that were a combination of the four parent faces, as shown in Figure 1. Each dimension was created by generating morphs containing different proportions of its two designated parent faces (in Figure 1: Parents A and B for the category-relevant dimension, and Parents C and D for the category-irrelevant dimension). The resulting dimensions contained a range or percentages of the second parent that was equal to 0%, 6%, 14%, 20%, 24%, 30%, 32%, 38%, 42%, 50%, 58%, 62%, 68%, 70%, 76%, 80%, 86%, 94%, and 100%. All levels of the category-relevant dimension were morphed with all levels of the category-irrelevant dimension such that the resulting two-dimensional morph included 50% from each of the one-dimensional morphs.

The second stimulus set was used in the identification training task, and it consisted of the face to be identified in later testing (Parent A of the category-relevant dimension) plus three unrelated faces from the original pool.

The third stimulus set was created using the Psychomorph software (http://users.aber.ac.uk/bpt/jpsychomorph/), to be used during the adaptation test. The full set of stimuli is shown in Figure 3, under the title “Target Stimulus.” First, the average face (0% morph in Figure 3) was obtained by morphing together all 30 male faces described above. Second, we obtained all other stimuli by morphing the average and target faces to varied degrees, going from 60% target face to −20% target face (or 20% anti-face) in 10% steps.

#### Procedure

##### Categorization training

Participants in the trained group were exposed to three 1-hr sessions of training in the categorization task shown in Figure 4, where the dots represent stimulus coordinates in the morph space, and the green line separates stimuli assigned to Categories A and B. This circular configuration of stimulus has been used in prior research on dimension differentiation (Folstein et al., 2012; Soto, 2019; Soto & Ashby, 2015, 2019), and was created to ensure that the distribution of stimuli does not suggest a dimensional structure in the task, which instead has to be learned from feedback (Goldstone & Steyvers, 2001). The sessions were run within a span of 3 days, and no more than two sessions were run on the same day. Consecutive sessions were separated by at least 1 hr and at most 25 hr. Each session consisted of nine blocks of 72 trials each, for a total of 648 trials. Some participants (see Participants section above) completed two 1.5 hr sessions of testing in a reverse correlation task. Results from those sessions are reported elsewhere (Soto, 2019). These participants received an additional 1 hr of categorization training before proceeding with the rest of the experiment.

At the beginning of each session, instructions were displayed on the screen indicating that the participant's task was to categorize the faces presented into two different groups (Category A or Category B) based purely on physical appearance. The instructions informed participants on the structure of each trial and how to report a categorization response. Participants were warned that during the early stages of the task, they would have to guess the correct answer but would eventually become more accurate as the experiment progressed.

Each session consisted of nine blocks composed of 72 trials, for a total of 648 trials per session. Each stimulus (36 per category) was presented once every block in a randomized manner. Throughout the session, there were voluntary breaks of 1 min between every block, which the participant could skip at any moment by pressing “enter” on the keyboard. If the participant allowed the entire minute to elapse, the next block would automatically begin when the break was over.

Each trial began with the presentation of a white cross in the middle of a black screen for 500 ms. Immediately afterward, a face stimulus was presented in the middle of the black screen until the participant pressed one of the two response buttons on the keyboard or a time deadline of 2 s was reached, whichever occurred first. To report whether the face shown belonged to either Category A or B, participants could press the keys D or K on their keyboard, which were relabeled as “A” and “B,” respectively. After every key press, participants received feedback on their accuracy. For correct responses, the word CORRECT was presented for 500 ms in green font color in the middle of the screen. For incorrect responses, the word INCORRECT was presented for 500 ms in red font color in the middle of the screen. Feedback was followed by a 1–s intertrial interval during which the monitor was completely black.

##### Identification training

Participants in both groups were exposed to a single 30-min session of identification training. The main goal of this task was to familiarize the participant with the identity of “Adam” in Figure 2 and train them to recognize his face from briefly presented images. This was achieved within the context of an identification task, which included three other faces, each with their corresponding name. Note that “Adam” corresponds to Parent A of the category-relevant dimension in Figure 1.

During the beginning of each session, instructions were displayed on the screen indicating that the task was to learn and identify the name of the face presented. The instructions included images of the four faces and their names: Adam, Sam, Kyle, and Luke. The instructions informed participants about the structure of each trial and how to report an identification response. Participants were warned to familiarize themselves well with the four different faces displayed and their names, and that faces would be presented very quickly during the task. Finally, they were told that during the early stages of the task, they would have to guess the correct answer but would eventually become more accurate as the experiment progressed.

The session consisted of 25 blocks of 20 trials each, for a total of 500 trials at completion of the session. Each face was presented five times per block in a randomized manner. Throughout the session, there were voluntary breaks of 1 min between every block, which the participant could skip at any moment by pressing “enter” on the keyboard. If the participant allowed the entire minute to elapse, the next block would automatically begin when the break was over.

Each trial began with the presentation of a white cross in the middle of a black screen for 500 ms, followed by the presentation of a face stimulus in the middle of the black screen for 200 ms. Immediately after this, participants could report whether the face shown was Adam, Sam, Kyle, or Luke, by pressing the keys A, S, K, or L on their keyboard, respectively. Participants had as much time as they needed to make their response. After every key press, participants received feedback on their accuracy. For correct responses, the word CORRECT was presented for 500 ms in blue font color in the middle of the screen. For incorrect responses, the word INCORRECT was presented for 500 ms in red font color in the middle of the screen. The given feedback was then followed by a 1-s inter-trial interval during which the monitor was completely black.

##### Adaptation testing

Participants in both groups were exposed to three 1-hr sessions of adaptation testing. Each session involved one of the three conditions presented in Figure 3: an identity adaptation condition, a category adaptation condition, and a control condition. Each session was broken into two parts. During the first part, participants underwent identification training, identical to that described in the previous section. However, this was only meant to serve as a reminder, so it consisted of only four blocks of 20 trials each, for a total of 80 trials.

Adaptation testing happened during the second part of the session, which had the goal of obtaining psychometric functions for the identification of “Adam” along the morph dimension presented in teal color in Figure 2 (i.e., going from anti-Adam to Adam and passing through the average face). Thus, all conditions involved the presentation of nine target stimuli varying along the anti-Adam/Adam morph dimension, each presented once in a block, with trials randomized within blocks. The two adaptation conditions involved 60 presentations of each stimulus (60 blocks of nine stimuli), whereas the control condition involved 120 presentations of each stimulus (120 blocks of nine stimuli).

The participant's task was simply to indicate in each trial whether the face presented was Adam or not. The instructions displayed informed participants of this task, indicated that strangers’ faces would also be presented, and instructed them to press the key “1” to indicate “Yes” and the key “2” to indicate “No.” Participants were also informed that they would no longer receive feedback about the correctness of their responses.

During the two adaptation conditions, the instructions also included information about the presentation of two different stimuli during each trial. Participants were instructed to look at the first face but completely ignore it for their responses during the task. They were also instructed to wait until the second response was quickly flashed on the screen, and then identify it as either Adam or not Adam.

During the two adaptation conditions, an adaptor was presented for 5 s before the target. In the identity adaptation condition, the adaptor presented was the anti-face of Adam (–100% in the morph dimension), whereas in the category adaptation condition, the adaptor presented was the second parent of the category-relevant dimension (Leo in Figure 2). No adaptor was presented in the control condition.

Trials involved the presentation of an adaptor for 5 s in the two adaptation conditions or no adaptor in the control condition. This was followed by 200 ms of a black screen and then the target stimulus was presented for 200 ms. Right after the target stimulus disappeared, the text “Was this Adam? Yes = 1, No = 2” was shown in the middle of the screen, and participants were able to record their response. A response was immediately followed by an intertrial interval of 500 ms. To prevent low-level adaptation aftereffects, the adaptor and target stimuli were presented with different sizes. The size of the adaptor image was 480×480 pixels, whereas the size of the target image was 384×384 pixels.

There were voluntary breaks of 1 min between every block, which the participant could finish at any moment by clicking “enter” on the keyboard. If the participant allowed the entire minute to elapse, the next block would automatically begin when the break was over.

##### Data analysis

**Figure 5.**
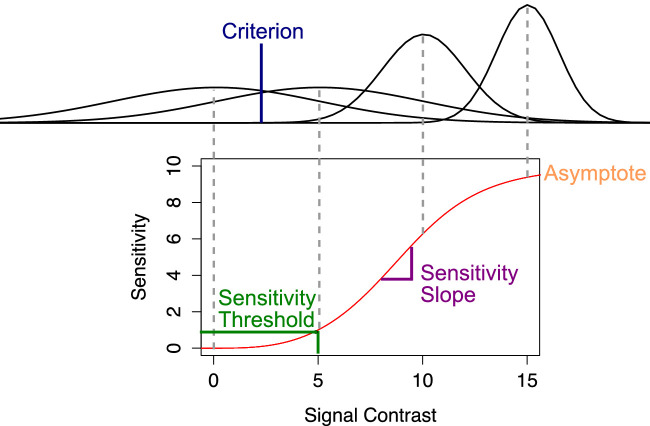
Explanation of the parameters of the detection-theoretic model fitted to the psychometric function data from Experiment 1. At the center of this model is the estimation of a sensitivity function, which links sensitivity in the *y*-axis and signal contrast *s* in the *x*-axis. Three parameters of this function are explained in the figure. At the top, it is possible to see the perceptual distributions assumed for four different levels of signal contrast. Sensitivity to their difference against zero is computed as the *d’* distance between the distributions. A decision criterion allows us to determine proportions of “yes” responses as a function of signal contrast, using the sensitivity function as an intermediate step.

We fit the data from each participant to a signal detection model of the psychometric function (Lesmes et al., 2015). At the center of this model is the estimation of a sensitivity function, shown in Figure 5, which describes sensitivity ($d^{'}$) as a function of the signal contrast s, which in our case is morph level, using the following equation:

(1) $$d^{'}\left(s\right)=\frac{\Lambda {\left(\frac{s}{\alpha }\right)}^{\beta }}{\sqrt{\left(\Lambda^{2}-1\right)+{\left(\frac{s}{\alpha }\right)}^{2\beta }}},$$

where β, Λ, and α represent the slope, upper asymptote, and position of the sensitivity function, respectively, as shown in Figure 5. The parameter α provides a direct estimate of a threshold (in units of s) corresponding to $d^{'}=1$. The sensitivity function from Equation 1 is linked to the psychometric function through the following equation:

(2) $$P\left({}^{''}yes^{''}|s\right)=\Phi \left(d^{'}\left(s\right)-\tau \right),$$

where $P\left({}^{''}yes^{''}|s\right)$ represents the probability of “yes” responses given a value of the morph dimension s, Φ represents the standard normal cumulative function and $\tau =\Phi^{-1}\left(1-FA\right)$ the decision criterion, with $\Phi^{-1}$ being the standard normal quantile function and FA the false alarm rate.

We fit this model to data using *R* v 3.2.1 (R Core Team, 2015) and the package *quickpsy* v. 0.1.4 (Linares & López-Moliner, 2016). The asymptotic sensitivity parameter Λ was fixed to a value of 10, which is similar to the values found by Lesmes et al. (2015). This corresponds to the assumption that the maximum discriminability between any two stimuli is not affected by adaptation or participant and helps in obtaining more precise estimates of all the other, more interesting parameters. The slope parameter β was limited to have a maximum value of 10, to avoid problems in the fits of some participants for whom this parameter would sometimes increase boundlessly to uninterpretable values. This did not affect the estimates for most participants, which were well below that limit.

After obtaining estimates of the parameters α (sensitivity threshold), β (sensitivity slope), and τ (decision criterion) from each participant's data, each of them was entered as a dependent variable in a separate 2 (Group) ×3 (Adaptor) mixed-effects analysis of variance (ANOVA) in *R*.

### Results and discussion

**Figure 6.**
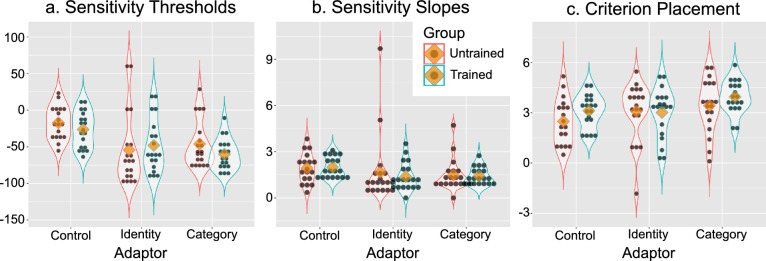
Results of Experiment 1. The *y*-axis in each plot represents the estimated parameter values. Black dots represent individual parameter estimates and yellow diamonds represent the group mean of the estimates. The red and blue violin shapes represent kernel density estimates of the distribution of parameter estimates for the untrained and trained groups, respectively.

The main results are presented in Figure 6. The most interesting results are presented in Panel a, which shows the estimated sensitivity thresholds. There seems to be an adaptation effect with both identity and category adaptors, evidenced by the drop in thresholds in those conditions compared to the control. More important, there is no evidence that categorization training modified such adaptation effect in any way. These results were confirmed by the ANOVA, which found a significant effect of adaptor, *F*(2, 104) = 4.23, *p* < .05, representing a global adaptation effect on sensitivity, whereas the main effect of group and the interaction were not significant. Post hoc pairwise comparisons (Bonferroni-corrected) revealed a significant difference between the control condition and both the identity and category adaptors (both *p* < .001), but no significant difference between these last two (*p* > .5).

Panels b and c show the estimated sensitivity slopes and criterion placement. These results are less interesting for the goals of our study, but they are presented because they allow us to determine whether adaptation and/or training have any effects on aspects of performance different from discriminability thresholds. The ANOVA results showed no significant effects in the analysis of sensitivity slopes (all *p* > .1), indicating that the increase in sensitivity as a function of stimulus value was unaffected by adaptation or training (Figure 6b). Similarly, the ANOVA showed no significant effects in the analysis of criterion placement (all *p* > .1), suggesting that the bias to report the target face was unaffected by adaptation or training.

A more commonly used measure of adaptation is simply the proportion of identification responses for an ambiguous target stimulus (e.g., the average face at 0% morphing) when presented alone, minus the same proportion when an adaptor is presented beforehand. We calculated such adaptation scores from our data, and we found adaptation scores above zero, but again no effect of adaptor, training, or their interaction in an ANOVA.

In sum, we found adaptation effects that were perceptual rather than decisional in nature, but categorization training had no effect on adaptation. That is, we found no evidence that categorization training reduces norm-based encoding of the parent of a category-relevant dimension or that it increases a link between the two parents of the category-relevant dimension.

However, Experiment 1 was limited in several ways. First, we tested only one parent of the category-relevant dimension. It is possible that categorization training had an effect on the other parent only. Second, we tested norm-based encoding by using the parent face as target and the anti-face as adaptor, but not the other way around. Finally, we focused on norm-based encoding, by presenting a sequence of target stimuli along the path of the average face (i.e., teal line in Figure 2), but we ignored the potential encoding of a completely novel category-relevant direction in space (i.e., red line in Figure 2).

Those are not limitations of the design of Experiment 1 per se but rather limitations imposed by the large amount of data that must be gathered in order to test all possibilities using psychometric functions, such as those obtained in this first experiment. The experiment was designed to obtain such functions and analyze them using a detection-theoretical model. Because we now know that our adaptation procedure produces perceptual effects with no contribution from decisional bias, and that similar results can be found using psychometric functions as well as simple adaptation scores, in the following experiments we reduced the amount of data recorded to a couple of ambiguous target stimuli, which allowed us to expand the experimental design considerably to more thoroughly test encoding of parent faces with and without categorization training.

## Experiment 2a

The results of Experiment 1 suggest that categorization training does not change the norm-based encoding of Parent A of the category-relevant dimension (see Figure 2). However, the experiment was limited in that it could only answer whether the encoding of that specific face identity changed after categorization. There was no testing of the encoding of Parent B of the category-relevant dimension. It is possible that categorization changes encoding of only one of the two parents, as the task can be solved by making decisions based on how similar each stimulus is to a single one of them (i.e., “Adamness” categorization).

More important, an open question is whether the direction in face space connecting the two parents of the category-relevant dimension, represented by the red line in Figure 2, is more strongly encoded after categorization training. From the point of view of the *dimension creation* hypothesis, a new perceptual representation of this category-relevant dimension should be constructed after categorization training. To be useful for the categorization task, this representation should contain information about the distance of a face from the category bound, which is equivalent to the categorization average shown in Figure 2. On the other hand, from the point of view of the *dimension enhancement* hypothesis, any changes in encoding of the two parents of the category-relevant dimension should be constrained to the directions in face space that were privileged before any training occurred, represented by the teal and orange lines in Figure 2. Experiment 1 found no evidence of such changes, but its limitations do not allow to completely rule out this hypothesis yet.

The present experiment was designed to more thoroughly explore how categorization training changes the encoding of the parents of the category-relevant dimension. To facilitate data collection, and given that the effect of adaptation was clearly restricted to sensitivity thresholds in Experiment 1, we evaluated the adaptation effect in specific ambiguous faces rather than in the full psychometric function.

The design involved all the faces presented in Figure 2. We first trained participants to identify Adam, Leo, and their anti-faces (which were renamed in the task). Then, during adaptation test, we presented both the global average and the category average as targets and asked participants to select which of the four identities was most likely being presented. In different blocks, we presented such targets without any adaptors or with any of the four identities as adaptors. We computed adaptation scores by taking the proportion of identification responses with and without an adaptor and then focused on three scores that were of particular interest. First, we computed an *anti-identity adaptation* effect, which was the average adaptation score observed for the parent faces when their anti-faces were presented as adaptors. Second, we computed an *identity adaptation effect*, which was the average adaptation score observed for the anti-faces when their corresponding parent faces were presented as adaptors. Finally, we computed a *category adaptation effect*, which was the average adaptation score obtained for each parent face when the other parent face was presented as adaptor. All three effects were recorded both at the global average and the categorization average, for a total of six combinations. Only two of these combinations were measured in Experiment 1, so the present experiment should provide a much richer data set to understand the effect of categorization training on face identity encoding.

As before, there were two groups of participants, one exposed to extensive training in the categorization task shown in Figure 1 and one without such training.

### Methods

#### Participants

Forty-eight students at Florida International University (78% female; ages between 18 and 30 years old, median = 22) participated in this experiment in exchange for a monetary compensation (U.S. $10/hour). Twenty-six participants were assigned to the trained group, and 22 participants were assigned to the untrained group. Nineteen participants (13 in the trained group, 6 in the untrained group) had previously participated in Experiment 1. Those participants retained their previous group assignment.

#### Stimuli

The stimulus set used in the categorization training task was the same as described for Experiment 1. The stimulus set used during adaptation testing included the same average identity as Experiment 1, as well as Parent A of the category-relevant dimension and its anti-face (Adam and anti-Adam in Figure 2). In addition, it included Parent B of the category-relevant dimension and its anti-face (Leo and anti-Leo in Figure 2), as well as the average morph between the two parents of the category-relevant dimension, labeled “Categorization Average” in Figure 2. All morphs were obtained using the procedures outlined in Experiment 1.

#### Procedure

##### Categorization task

All procedures were exactly the same as those described for Experiment 1, with the exception of the number of blocks in the categorization session and the number of categorization training sessions completed by the trained group participants.

Participants who had previously participated in Experiment 1 had already completed three to four categorization-training sessions. Therefore, these returning participants completed one 30-min categorization reminder session before completing the following test. The instructions and setup of the task were identical to the categorization task in Experiment 1, but the session consisted of only five blocks rather than nine.

Participants in the trained group who had not previously participated in Experiment 1 completed three 1-hr sessions of the categorization task, as described for Experiment 1. Like the returning participants, these newly recruited participants completed one 30-min categorization reminder session before completing the following test.

##### Adaptation testing

Participants in all groups were exposed to one 1-hr session of adaptation testing. Each session was divided into two parts.

During the first part of each session, participants were trained in an identification task. Four faces were included, corresponding to Adam, Leo, and their anti-faces displayed in Figure 2. Anti-Adam was given the name Scott, whereas anti-Leo was given the name Kurt.

At the beginning of this identification training, instructions were displayed on the screen indicating that the participant's task was to learn and identify the names of four faces. The instructions included images of the four faces and their names: Adam, Scott, Kurt, and Leo. The instructions informed participants on the structure of each trial and how to report an identity response. Participants were warned to familiarize themselves well with the four faces displayed and their names, and that faces would be presented very quickly during the task. They were also warned that during the early stages of the task, they might have to guess the correct answer for each face image, but through feedback, they would eventually become more accurate as the experiment progressed.

This identification training part consisted of 25 blocks of 4 trials each, for a total of 100 trials. Each face was presented once every block in a randomized manner.

Each trial began with a black screen for 200 ms, followed by the presentation of a face stimulus in the middle of the black screen for 200 ms. Immediately after this, the following text was presented: “Who was this? Adam = A, Scott = S, Kurt = K, Leo = L,” and participants were allowed to record their response. After a key press, participants received feedback on their accuracy. For correct responses, the word CORRECT was presented for 1 s in blue font color in the middle of the screen. For incorrect responses, the word INCORRECT was presented for 1 s in red font color in the middle of the screen. The given feedback was then followed by a 500-ms intertrial interval during which the monitor was completely black.

During the second part of the session, participants underwent adaptation testing. Each trial during this testing was structured as described for adaptation testing in Experiment 1. Here, however, both the adaptors and target stimuli were different from those used in Experiment 1.

There were five blocks of testing, which were identical except for the adaptor used. Each of the four stimuli presented during identification training was used as adaptors in different blocks, and there was one baseline block in which no adaptor was presented. The baseline block was always presented first in the sequence, and the other blocks were presented in random order.

Each testing block began with seven blocks of identification training, for a total of 28 trials. These were presented just as a reminder of the correct assignment of faces to response keys. In adaptation blocks, this was followed by instructions. During the first adaptation block (i.e., immediately after finishing the baseline block), detailed instructions were provided. Participants were told that two different faces would be presented during each trial, with the first face being presented for a long time and the second face briefly flashed on the screen. Participants were instructed to look at the first face but completely ignore it for their responses during the task. On the other hand, they were instructed to identify the second face as Adam, Scott, Kurt, or Leo, by using the response keys previously learned. In subsequent adaptation blocks, more brief instructions were presented, simply reminding participants that now they would see trials with one face following another and that they should only look at the first face but identify the second one.

Each block was composed of six sub-blocks of 10 trials each: 5 using the global average as a target and 5 using the category average as a target. Trials were randomized within these sub-blocks. In total, each target was presented 30 times in each block.

There were voluntary breaks of 1 min between every block in which the participant could finish at any moment by clicking “enter” on the keyboard. If the participant allowed the entire minute to elapse, the next block would automatically begin when the break was over.

##### Data analysis

Performance criteria to include data in the main analysis were the same as in the previous study, that is, 65% correct in the categorization task, and 85% correct in the main identification task during testing.

Adaptation scores were computed by taking the proportion of identifications of a given face during blocks in which a specific adaptor was presented and subtracting the proportion of identifications of the same face during baseline blocks. These scores were used to compute three different effect scores per participant. First, an *anti-identity adaptation* effect was computed as the average adaptation score observed for the parent faces (i.e., involving identification responses for the parent faces) when their anti-faces were presented as adaptors. Second, an *identity adaptation effect* was computed as the average adaptation score observed for the anti-faces (i.e., involving identification responses for the anti-faces) when their corresponding parent faces were presented as adaptors. Finally, a *category adaptation effect* was computed as the average adaptation score obtained for each parent face (i.e., involving identification responses for a given parent face) when the other parent face was presented as adaptor. All three effect scores were separately computed for trials in which the global average and the categorization average were presented as target stimuli. Separate data analyses were performed for each combination of target and adaptation effect.

Differences between groups were tested using a one-tailed independent-samples *t* test. We tested whether categorization training reduced norm-based encoding of identity, as evidenced by a reduced anti-identity adaptation effect or a reduced identity adaptation effect. We tested whether categorization training increased categorical encoding of identity, as evidenced by an increased category adaptation effect. A one-sample *t* test (one-tailed) was used to test whether each adaptation effect was significantly higher than zero.

### Results and discussion

Using the preset criteria for inclusion into the main analysis, 9 participants were eliminated from each group, resulting in the inclusion of 13 participants in the untrained group and 17 in the trained group. Performance in the categorization training task was 81.94% correct on average (*SD* = 4.69%). Performance in the identification task was 92.14% correct on average (*SD* = 4.42%).

**Figure 7.**
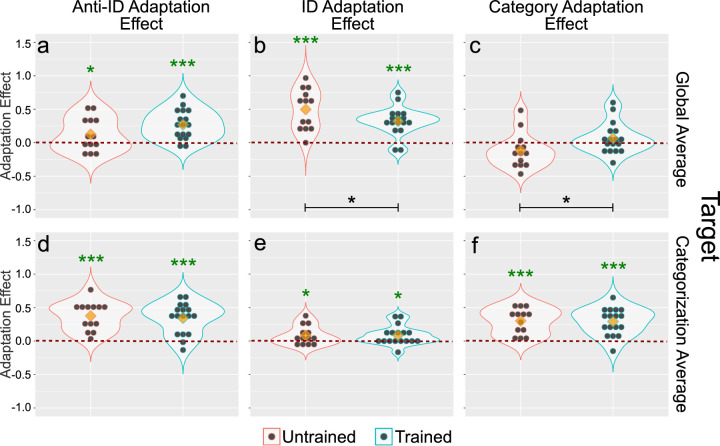
Results of Experiment 2. Each column represents a different adaptation effect score, and each row represents a different target face presented during the adaptation test. Blue and red outlines represent data from the trained and untrained groups, respectively. Black dots represent individual participant scores, yellow diamonds represent the mean of each subset of the data, and the outlines represent kernel density estimates obtained from such subsets. The red dotted line represents no adaptation effect. Green asterisks represent a significant adaptation effect, and black asterisks represent a significant difference between groups in the adaptation effect, with ${}^{*}p<.05$, ${}^{**}p<.01$, and ${}^{***}p<.001$.

The top row of plots in Figure 7 (Panels a—c) shows the results of the analysis when the target stimulus presented was the global average. Figure 7a shows the anti-identity adaptation effect, in which anti-identities were presented as adaptors and the adaptation effect was measured for their corresponding identities. The adaptation effect was significant both in the trained group, *t*(16) = 5.07, *p* < .001, and in the untrained group, *t*(12) = 1.81, *p* < .05. Categorization training did not reduce the norm-based encoding of identity, as it would be evidenced by a reduced adaptation effect, *t*(28) = −1.68, *p* > .5. If anything, there is evidence of an increased adaptation effect, with an effect that was slightly higher in the trained group than in the untrained group. This reversed effect was also not significant (one-tailed *p* = .052). These results are consistent with those obtained in Experiment 1, revealing an overall adaptation effect but no effect of categorization training on norm-based encoding of the parent identities.

Figure 7b shows the identity adaptation effect, in which parent identities were presented as adaptors and the adaptation effect was measured for their corresponding anti-identities. The adaptation effect was again significant both in the trained group, *t*(16) = 6.14, *p* < .001, and in the untrained group, *t*(12) = 5.93, *p* < .001. In addition, categorization training did significantly reduce the adaptation effect in this case, *t*(28) = 1.84, *p* < .05. This is surprising, as this identity adaptation effect should tap into the same mechanisms as the anti-identity adaptation effect measured above and in Experiment 1, which were not influenced by training. We discuss this asymmetrical result in more detail in the General Discussion, but for now, the important conclusion from this test is that categorization training does influence the norm-based encoding of face identity.

Figure 7c shows the category adaptation effect, in which a parent identity was presented as adaptor and the adaptation effect was measured for the other parent identity. Neither adaptation effect was significant (*p* > .05). However, the category adaptation effect was slightly enhanced after categorization training, being on average below zero before training, but above zero after training. This difference was significant, *t*(28) = 2.03, *p* < .05.

Overall, these results show a clear effect of categorization training on encoding of face identity, revealed by a reduction in the adaptation effect that used the category parents as adaptors and an increase in the category adaptation effect.

The bottom row of plots in Figures 7d–f shows the results of the analysis when the target stimulus presented was the categorization average. Figure 7d shows the anti-identity adaptation effect, in which anti-identities were presented as adaptors and the adaptation effect was measured for their corresponding identities. The adaptation effect was again significant both in the trained group, *t*(16) = 6.06, *p* < .001, and in the untrained group, *t*(12) = 6.40, *p* < .001. However, there was no effect of categorization training on this adaptation effect, *t*(28) = .39, *p* > .5.

Figure 7e shows the identity adaptation effect, in which parent identities were presented as adaptors and the adaptation effect was measured for their corresponding anti-identities. The adaptation effect was significant both in the trained group, *t*(16) = 2.57, *p* < .05, and in the untrained group, *t*(12) = 2.13, *p* < .05, but their difference was again not significant, *t*(28) = .08, *p* > .5.

Figure 7f shows the category adaptation effect, in which a parent identity was presented as adaptor and the adaptation effect was measured for the other parent identity. Unlike the results shown in Figure 7c, here the category adaptation effect was significant both in the trained group, *t*(16) = 5.90, *p* < .001, and in the untrained group, *t*(12) = 5.63, *p* < .001. However, there was no increase in the strength of this effect with categorization training, *t*(28) = .13, *p* > .5.

In sum, the results measured at the global average were consistent with those from Experiment 1, in that there was no effect of categorization training on the anti-identity adaptation effect. On the other hand, categorization training did reduce the identity adaptation effect and, unlike in Experiment 1, also increased the category adaptation effect. These results suggest that categorization training does change the norm-based encoding of face identity.

On the other hand, categorization training had no effect in any adaptation effect measured at the categorization average. That is, there was no evidence suggesting that the category-relevant dimension becomes a preferred direction in the encoding of the parent faces.

The overall pattern of results is better explained by the *dimension enhancement* hypothesis, which proposes that categorization training only modifies already existing representations, than by the *dimension creation* hypothesis, which proposes that categorization training creates novel stimulus representations. In the General Discussion, we present a formal model implementing the dimension enhancement hypothesis, which explains the pattern of significant changes produced by categorization training as shown in Figures 7a–c.

## Experiment 2b

Prior research has shown that categorization training increases the invariance of the category-relevant dimension (or its “separability”; see Ashby & Soto, 2015; Soto et al., 2017) to changes in category-irrelevant morphed face dimensions (Soto & Ashby, 2015; Soto, 2019). Experiment 2b was a simple extension of Experiment 2a, aimed at using adaptation procedures to explore whether categorization training increases invariant encoding of category-relevant faces.

In particular, we used a test that was previously used to determine whether the encoding of face identity is invariant to changes in facial expression and vice versa (e.g., Ellamil et al., 2008; Fox & Barton, 2007; Fox et al., 2008; Webster et al., 2004). In this test, which here we call *cross-adaptation*, stimuli vary along an adaptation dimension and a modulatory dimension. For example, one might be interested in the encoding of face identity, as in the previous experiments. In that case, adaptor and target stimuli differ in their level along some identity dimension, and participants report the perceived identity of the target. The idea behind cross-adaptation is to test whether the observed adaptation effects generalize across changes in the modulatory dimension. For example, Fox et al. (2008) compared the strength of the identity aftereffect when the expression shown by the adaptor and target faces was congruent (e.g., both angry) versus when it was incongruent (e.g., one angry and one fearful). If encoding of identity is invariant to changes in expression, then the identity aftereffect should be the same in congruent and incongruent trials, showing strong cross-adaptation across expression. On the other hand, if encoding of identity is not invariant to changes in expression, then the identity aftereffect should be stronger in congruent than in incongruent trials, showing poor cross-adaptation across expression.

Here, we used the cross-adaptation test to determine whether, with categorization training, encoding of parent faces of the category-relevant dimension becomes more invariant to changes in the category-irrelevant dimension. The same participants from Experiment 2a were exposed to a second adaptation test. This test was similar to the first, with the exception that adaptors and targets were morphed with the parents of the category-irrelevant dimension (Parents C and D in Figure 1). Each participant was exposed to adaptors having a single level of the category-irrelevant dimension (counterbalanced across participants), but to targets with both levels of the category-irrelevant dimension. That is, in different trials, the level of the category-irrelevant dimension could be congruent or incongruent between adaptor and target. This allowed us to test the effect of congruency on all the adaptation effects previously introduced in Experiment 2a, while it also served as a way to test the reliability of the effects observed in that experiment.

### Methods

#### Participants

Participants were the same as those from Experiment 2a. One participant from each group did not return for this second test, producing 21 participants in the untrained group and 25 participants in the trained group.

#### Stimuli

The stimuli used during categorization training were the same as those used in Experiments 1 and 2.

The stimuli used during the cross-adaptation test were created by morphing all the stimuli from the previous experiment with the parents of the category-irrelevant dimension (Parents C and D in Figure 1), where the contribution of the irrelevant dimension was 30%. This percentage was chosen so that the change in the irrelevant dimension was perceptually obvious, but to avoid a change so large that it would make identification of the parents of the category-relevant dimension too difficult for participants to master quickly. The reason to avoid making identification too difficult is that, as can be seen from the examples in Figure 1, it is quite difficult to discriminate changes in multidimensional morphed faces, and many participants can never master categorization and identification tasks involving such stimuli.

#### Procedure

##### Categorization task

All procedures were exactly the same as those used in Experiment 2.

##### Cross-adaptation test

All procedures were exactly the same as those used for Experiment 2, with the following exceptions.

The first part of the session consisted of 10 blocks in which each of the four stimuli identified as Adam, Scott, Kurt, or Leo were presented once, for a total of 40 training trials. The task was the same as in Experiment 2, with the exception that the four stimuli used were morphed with 30% of the average face, in order to train participants in a more difficult identification task than what they encountered during Experiment 2a. The reason behind this was that all stimuli presented during the cross-adaptation test would only include 70% of the relevant face.

Adaptation testing proceeded as in Experiment 2a, but each adaptor was presented in a single level of the irrelevant dimension, either Level 1 or Level 2 (corresponding to 30% of morphing of the adaptor with the two parents of the category-irrelevant dimension, as explained above in the Stimuli section, with each parent representing one level). The assignment of level of the irrelevant dimension was counterbalanced across participants within each group. Each test block was subdivided into six smaller blocks, each with five presentations of four different target faces, resulting from the combination of the same target faces as in Experiment 2 (global average and categorization average; see Figure 2), each at Levels 1 and 2 of the irrelevant dimension. Each target was shown 30 times per block for a total of 120 trials per block.

##### Data analysis

Data analysis proceeded as in Experiment 2a, but adaptation effect scores were entered into a 2 (Group: trained vs. untrained) ×2 (Congruency: congruent vs. incongruent level of the irrelevant dimension between adaptor and target) mixed-effects ANOVA, rather than to a *t* test.

### Results and discussion

Using the same preset criteria for exclusion as in prior experiments, 9 participants were eliminated from the untrained group and 11 from the trained group. This resulted in 12 participants in the untrained group and 14 in the trained group. Performance in the categorization training task was 82.04% correct on average (*SD* = 5.67%). Performance in the identification task was 90.58% correct on average (*SD* = 2.91%).

**Figure 8.**
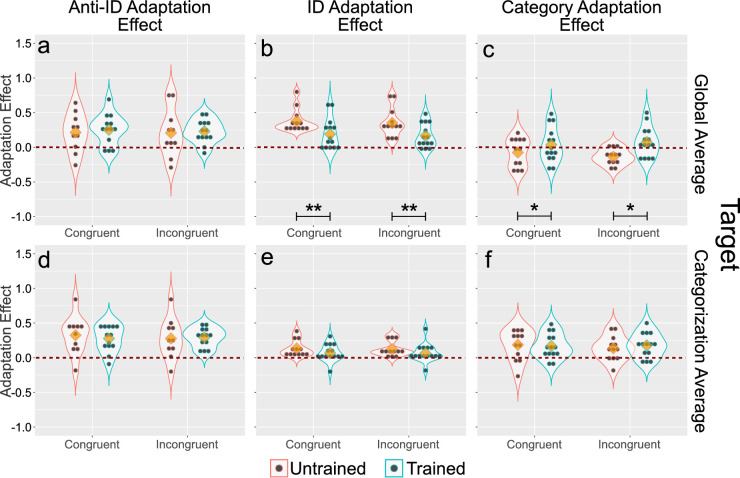
Results of Experiment 2b. Each column represents a different adaptation effect score, and each row represents a different target face presented during the adaptation test. Blue and red outlines represent data from the trained and untrained groups, respectively. Data are presented separately in each plot for congruent (same level of the irrelevant dimension in adaptor and target stimuli) and incongruent (different level of the irrelevant dimension in adaptor and target stimuli) trials. Black dots represent individual participant scores, yellow diamonds represent the mean of each subset of the data, and the outlines represent kernel density estimates obtained from such subsets. The red dotted line represents no adaptation effect. Black asterisks represent a significant difference between groups in the adaptation effect, with ${}^{*}p<.05$, ${}^{**}p<.01$, and ${}^{***}p<.001$.

The top row of plots in Figures 8a–c shows the results of the analysis when the target stimulus presented was the global average. Figure 8a shows the anti-identity adaptation effect. It can be seen that congruency of the adaptor and target along the irrelevant dimension had little influence on this effect, which resulted in a nonsignificant effect of Congruency, *F*(1, 40) = .49, *p* > .1, and a nonsignificant Group × Congruency interaction, *F*(1, 40) = .01, *p* > .5. As in the previous experiment, categorization training slightly increased the adaptation effect, but the effect of group was not significant, *F*(1, 40) = .16, *p* > .5.

Figure 8b shows the identity adaptation effect. Again, congruency of the adaptor and target along the irrelevant dimension had little influence, resulting in a non-significant main effect of the congruency factor, *F*(1, 46) = .01, *p* > .5, and a nonsignificant Group × Congruency interaction, *F*(1, 46) = .00, *p* > .5. On the other hand, categorization training significantly decreased the adaptation effect, *F*(1, 46) = 9.25, *p* < .005, which replicates the finding from the previous experiment.

Figure 8c shows the category adaptation effect. Once again, there was no significant effect of congruency, *F*(1, 46) = .57, *p* > .1, or the Group × Congruency interaction, *F*(1, 46) = 1.16, *p* > .1. However, the category adaptation effect was slightly enhanced after categorization training, a difference that resulted in a significant effect of group, *F*(1, 46) = 6.77, *p* < .05. As in Experiment 2a, the category adaptation effect is slightly below zero in the untrained group and slightly above zero in the trained group.

Overall, these results mirror those of Experiment 2a in showing a clear effect of categorization training on encoding of face identity, revealed by a reduction in the identity adaptation effect, and an increase in the category adaptation effect. On the other hand, there was no evidence of an effect of congruency of the adaptor and target along the irrelevant dimension.

The bottom row of plots in Figures 8d–f shows the results of the analysis when the target stimulus presented was the categorization average. Figure 8d shows the anti-identity adaptation effect. No effects were significant in the ANOVA (group: *F*(1, 40) = .00, *p* > .5; congruency: *F*(1, 40) = .00, *p* > .5; Group × Congruency: *F*(1, 40) = .19, *p* > .5). Similarly, Figure 8e shows the identity adaptation effect, and again, no effects were significant in the ANOVA (group: *F*(1, 46) = 2.74, *p* > .1; congruency: *F*(1, 46) = .01, *p* > .5; Group × Congruency: *F*(1, 46) = .01, *p* > .5). Finally, Figure 8f shows the analysis of the category adaptation effect, where again no effects were significant (group: *F*(1, 46) = .06, *p* > .5; congruency: *F*(1, 46) = .92, *p* > .1; Group × Congruency: *F*(1, 46) = .58, *p* > .1).

In sum, the results regarding the effect of categorization training on adaptation exactly reproduce those found in the previous experiment, showing that they can be reliably obtained across different stimulus sets. In that sense, the results of this experiment can be considered successful.

On the other hand, we found cross-adaptation across changes in the irrelevant dimension in both groups, indicating that either encoding of the category-relevant parent identities is invariant to changes in the category-irrelevant identity, or that the contribution of the category-irrelevant dimension to the test stimuli was not large enough to detect a drop in the adaptation effect. In favor of the invariance hypothesis, the contribution of the category-irrelevant dimension to the test stimuli was large enough to be easily perceived. One possibility is that the extensive identity training received by our participants (they were trained in both Experiments 2a and 2b, with the task made difficult through morphing in the latter experiment) in both groups produced invariant identity representations. In favor of the insensitive tests hypothesis, a body of work suggests that novel morphing dimensions are not invariant but encoded in an integral/configural manner (Blunden et al., 2015; Folstein et al., 2012; Goldstone & Steyvers, 2001; Soto & Ashby, 2015; Soto, 2019). However, none of those tests involved adaptation testing, and as research from the interaction between face identity and expression shows, the results of cross-adaptation tests are not always in line with the results from other tests of invariance. More research will be necessary to decide between these two possibilities, but at the very least, the results of the present experiment served to demonstrate the reliability of the pattern of results observed in Experiment 2a.

## General discussion

Here, we used face adaptation aftereffects to explore how categorization training changes the encoding of face identities at the extremes of the category-relevant dimension. Most previous work on perceptual effects of categorization training have favored an interpretation of results in terms of dimension creation or differentiation (Goldstone & Steyvers, 2001; Folstein et al., 2013, 2015; Schyns et al., 1998), which assumes the creation of novel representations of the category-relevant and category-irrelevant dimensions that are used to perceptually organize stimuli beyond the original training task. We contrasted this to an alternative interpretation in terms of *dimension enhancement*, meaning that categorization training produces alterations in already existing visual representations, simply enhancing the prelearning selectivity of populations of neurons that happen to provide useful information for the categorization task (Brants et al., 2016).

Surprisingly, our results seemed to support the less popular *dimension enhancement* hypothesis. In Experiment 1, we failed to see any effect of categorization training on identity aftereffects, when such aftereffects were tested by presenting anti-identities as adaptors and measuring identification of the parents of the category-relevant dimension. Experiments 2a and 2b reproduced this lack of an effect of categorization training, but they also showed two reliable effects of such training: (a) Categorization training decreases identity aftereffects measured by presenting the parents of the category-relevant dimension as adaptors and measuring identification of their anti-faces, and (b) categorization training increases identity aftereffects measured by presenting one parent of the category-relevant dimension as adaptor and measuring identification of the other parent. Importantly, all these effects were observed when the global average face was used as a target in the adaptation design (gray circle in the middle of Figure 2), which could be explained as resulting from modification of the already existing norm-based encoding of face identities. When adaptation was measured using the categorization average as a target (red circle in Figure 2), categorization training produced no effects. This suggests that categorization training does not promote encoding the category-relevant dimension as a preferred direction in face space but rather modifies encoding of already preferred directions that pass through the face average.

In the following section, we present a mechanistic explanation of the pattern of results observed in the present experiments, which is essentially a formalization of the *dimension enhancement* hypothesis within the theoretical framework of face-space encoding.

### A working hypothesis

**Figure 9.**
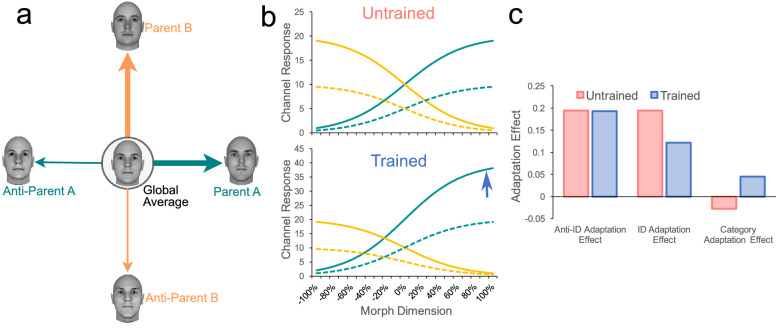
A model implementing the *dimension enhancement* hypothesis as it would be applied to the present study. From the point of view of this hypothesis, the only effect of categorization training involving a category-relevant dimension joining Parent A and B (Panel a) is an enhancement in the already existing encoding of those specific identities. As shown in Panel b, this is achieved by simply assuming that categorization training scales up the response of channels that are sensitive to the presence of Parent A or B. By assuming that adaptation scales down responses in the affected channels (dotted lines in Panel b), the model makes predictions that match the results observed in Experiments 2a and 2b, when adaptation was tested at the global average face. These predictions are shown in Panel c and should be compared to results shown in Panels a to c of Figures 7 and 8.

Figure 9 shows a plausible mechanistic explanation for the effects of categorization training observed in the present experiments (for mathematical details of the model, see the Appendix). Note first that we follow here previous authors in modeling the response of opponent channels to stimuli changing along a single dimension (going from a parent face to its anti-parent; see McKone et al., 2014; Jeffery et al., 2018), although in reality, such dimension would be embedded in a multidimensional face space (Valentine et al., 2016). The first channel, represented by the solid teal curves, responds more strongly to faces that are more similar to the parent identity. The second channel, represented by the solid yellow curves, responds more strongly to faces that are more similar to the anti-parent identity. A wealth of psychophysical (e.g., Leopold et al., 2001; McKone et al., 2014; Rhodes & Jeffery, 2006), neurophysiological (e.g., Giese & Leopold, 2005; Freiwald et al., 2009; Leopold et al., 2006), and neuroimaging (e.g., Carlin & Kriegeskorte, 2017; Loffler et al., 2005) data support such coding for face identity (but see Ross et al., 2013). However, we assume that a more complete model would involve explicit modeling of the full face space and the multidimensional tuning of many channels distributed in such space (e.g., Giese & Leopold, 2005; Ross et al., 2013). In addition, we assume here a single norm, because all our stimuli correspond to Caucasian male faces (as in previous studies in this area of research; Goldstone & Steyvers, 2001; Soto & Ashby, 2015, 2019; Soto, 2019). However, we must note that there is evidence for the existence of different norms for different genders (Rhodes et al., 2011) and races (Jaquet et al., 2008).

The main novel hypothesis that we propose is quite simple, and it is schematically represented in Figure 9a. The idea is that the only effect of categorization training is an enhancement in the representation of the identities at the extremes of the category-relevant dimension. As represented in the figure, face encoding after categorization training would remain norm-based, just as before training, but the system becomes biased to detect shape features of Parents A and B of the category-relevant dimension. This can be achieved by simple scaling of the responses of the population of neurons whose preferred stimuli are those face identities. This is shown in Figure 9b, where one of the morphed dimensions from Figure 9a is encoded using two channels with opponent stimulus preference. As shown by the blue arrow, the only effect of categorization training is a scaling up of the response of the first channel.

The dotted curves shown in Figure 9b represent the effect in a channel's response of continued presentation of its preferred stimulus, as it happens during adaptation. We make the simple assumption that adaptation produces a scaling down in the response of the channel, which is proportional to its nonadapted response. This simple divisive scaling of channel responsiveness is only one of multiple neural mechanisms that could be behind adaptation (Kohn, 2007; Solomon & Kohn, 2014; Vogels, 2016), with others including subtractive reduction, shifts in the tuning curve (Kohn, 2007), and normalization (Solomon & Kohn, 2014; Vogels, 2016). However, most of the direct evidence for such mechanisms comes from studies in early visual cortex using simple visual stimuli; it remains unknown which of those mechanisms acts in the case of face adaptation. In addition, divisive scaling has been widely used in prior modeling work (e.g., McKone et al., 2014; Jeffery et al., 2018; Giese & Leopold, 2005; Ross et al., 2013; Series et al., 2009) to explain a variety of psychophysical (McKone et al., 2014; Jeffery et al., 2018; Ross et al., 2013; Series et al., 2009) and neuroimaging (Alink et al., 2018) observations.

These very simple assumptions, plus a decision rule based on the ratio rule (Luce, 1959), are all that is necessary to explain the effects of categorization training observed when the global average was used as a target (0% morphing in Figure 9b). As shown in Figure 9c, the model reproduces the lack of an effect of categorization training on the anti-identity adaptation effect, observed in all our experiments, but the presence of an effect of categorization training on the identity adaptation effect observed in Experiments 2a and 2b. To understand these effects, one must first note that the adaptation effects are measured relative to a nonadapted baseline of responding. For example, in the top panel of Figure 9b, baseline responding to the global average (the 0% point) does not favor either the parent or anti-parent identities, as both of them have the same value (solid lines, which cross at 0%). After adaptation to the anti-parent, there is a drop in responding in the corresponding channel (dotted yellow curve), resulting in a bias to identify the parent at the global average. Now switch your attention to the bottom panel of Figure 9b. Here, baseline responding is already biased toward identification of the parent face (compare solid lines at 0%). After adaptation to the anti-parent, the addition to this bias produced by the drop in anti-channel responding is equivalent to that observed in the untrained condition.

Something different happens when the parent is the adaptor and the effect is measured for identification of the anti-parent. Without training, the situation is the same as before. However, after training, there is a baseline bias to identify the average as the parent, and the effect of adaptation is simply to get rid of that bias, rather than creating a new bias toward identifying the anti-parent. Thus, the adaptation effect is smaller than in the untrained condition.

Finally, the model reproduces a below-zero category adaptation effect in the untrained group and above-zero adaptation effect in the trained group, also observed in both Experiments 2a and 2b. In the untrained group, adaptation to one of the parent's simply produces a strong bias to choose its anti-parent. This produces a general drop in all other choices, including the choice of the second parent of the category-relevant dimension. This is how the category adaptation effect ends up being negative in the untrained group. The same process occurs in the trained group, but now categorization has created a baseline bias toward identifying either of the parents of the category-relevant dimension. Thus, when responding in the channel preferring one of the parent's drops, responding in the channel preferring the other parent is now relatively strong, producing an adaptation effect. However, the effect is not very large, as the tendency in norm-based encoding is to often choose the anti-parent when a parent has been adapted.

If the *dimension enhancement* hypothesis presented in Figure 9 is correct, then one consequence is that categorization training should increase the baseline proportion of identifications of the parent faces and reduce the baseline proportion of identifications of the anti-parent faces. We performed a 2 (Group) ×4 (Choice: Parent A, Parent B, Anti-parent A, and Anti-parent B) mixed-effects ANOVA with proportion of choices during baseline as dependent variable and found a significant interaction effect, *F*(3, 144) = 4.453, *p* < .01. The interaction was due to an increase in responding to Parent A and a decrease in responding to Anti-parent A as a result of categorization training, partially confirming the prediction of the model presented in Figure 9, but suggesting that categorization training might affect the representation of one of the parent faces more than the other. Related asymmetrical results have been observed before (Soto, 2019), and they suggest that what is learned during categorization training is to extract evidence for the presence of one of the parents and choose that parent when the evidence is high and the other parent when the evidence is low (i.e., solving the categorization task based on the “Adamness” of the presented faces). The model results presented in Figure 9 assume equivalent effects in the encoding of both parents of the category-relevant dimension, so clearly a better implementation of the model and a more systematic exploration are necessary.

We must highlight that the results in Figure 9 are presented as a proof of concept rather than as a full-fledged model of dimension differentiation. Such a theory would require additional details in the model (e.g., modeling neural noise and a choice rule capable of handling such noise). Ideally, the theory would be able to explain additional results in the literature. For that to happen, the model might require a better formalization of representations in face space than what is presented in Figure 9. That is, the model presented in the figure works by simply focusing on a couple of dimensions embedded within a large multidimensional space of face features. If such space were explicitly modeled, it would allow, for example, generating predictions not only about the results at the global average, but also at the category average (i.e., the bottom rows of Figures 7 and 8, which were not simulated here). There are multiple ways in which a multidimensional face space has been modeled in the previous literature. For example, one can use a relatively arbitrary face space (Ross et al., 2013), a space created through principal component analysis of actual face images (Turk & Pentland, 1991), or a space of face-selective units at the end of a hierarchical feedforward model of the ventral visual stream (Giese & Leopold, 2005; Jiang et al., 2006). Multiple channels should be modeled rather than only two, with each channel's tuning function, as well as internal noise, defined within the high-dimensional face space (Giese & Leopold, 2005; Ross et al., 2013). Finally, implementing the *dimension enhancement* hypothesis in a multidimensional space would be more complex than in the simple model presented in Figure 9, with the scaling mechanism influencing multiple channels distributed around the parent faces. The development of a full computational theory is outside the scope of the current study, and the model presented in Figure 9 should be taken only as a working hypothesis.

However, at least intuitively, this hypothesis seems able to explain other results in the literature. For example, if the two parents of the category-relevant dimension are perceived more easily from ambiguous stimuli, this would explain the observed increase in discriminability along the category-relevant dimension, or acquired distinctiveness (Folstein et al., 2012, 2013, 2014; Goldstone & Steyvers, 2001; Van Gulick & Gauthier, 2014). This facilitated perception might also interfere with the extraction of information about other, category-irrelevant features, resulting in acquired equivalence. A combination of both effects would explain an increase in invariance of the category-relevant dimension (Soto & Ashby, 2015; Soto, 2019). The *dimension enhancement* hypothesis would also explain why categorization training does not dramatically change templates for face identification but rather seems to strengthen features of already existing identity representations, as shown by reverse correlation (Soto, 2019).

Category learning is usually faster and more generalizable when the category bound aligns with a preexisting dimensional structure in the stimuli. Morphed face dimensions lack such dimensional structure, but they appear to acquire it on-the-fly during training in a categorization task (Soto & Ashby, 2019). The *dimension enhancement* hypothesis would explain why such learning of representations that support categorization is so fast, as it would require only the modification of already existing representations rather than the creation of new representations.

The results presented here and the model sketched in Figure 9 are in line with the functional MRI results reported by Brants et al. (2016). These authors trained a classifier to decode novel object categories using patterns of activity from high-level visual cortex obtained before categorization training and tested this pretrained classifier with patterns of activity obtained after categorization training. The *dimension creation* hypothesis predicts that generalization from pre- to post-training data should be difficult. On the other hand, the *dimension enhancement* hypothesis predicts strong generalization from pre- to post-training data, which is exactly what Brants and colleagues found.

Our conclusion is also in line with an assumption of the face-space theory of face representation, which proposes that dimensions in face space are scaled to optimize discrimination based on experience (Valentine et al., 2016). Originally, this assumption was used to explain the other-ethnicity effect, in which people are worse at identifying faces from other ethnicities than faces from their own ethnicity, probably due to a difference in experience with each group. One way in which the scaling of dimensions in face space might occur is as a result of categorization training. Categorization might promote rescaling of dimensions that enhances between-category discriminability and reduces within-category discriminability, especially if it is not accompanied by other tasks requiring more fine-grained identity discrimination.

### Brain mechanisms of dimension differentiation

As indicated earlier, it is commonly assumed that representations such as those shown in Figure 9 are stored in face-selective areas within inferior temporal cortex, at the latest stages of processing in the visual ventral stream. An important question then is whether the current literature on learning-related changes within inferior temporal cortex provides clues about the neurocomputational mechanisms underlying dimension differentiation and whether they agree with a dimension enhancement hypothesis.

There is evidence that, when stimuli vary along already existing separable dimensions, neurons in inferior temporal cortex become more selective to features of complex stimuli that are diagnostic of category membership, including faces (De Baene et al., 2008; Sigala & Logothetis, 2002; see also Ester et al., 2020). This is in line with the dimension enhancement hypothesis, but an important problem is that morphed dimensions, such as those used here and in previous studies on dimension differentiation, are known to be integral (Blunden et al., 2015; Goldstone & Steyvers, 2001; Soto & Ashby, 2015).

Early studies using such integral morphed dimensions found small effects of categorization on tuning of inferior temporal cortex neurons, which could be explained as resulting from the neurons’ selectivity to visual shape and simple familiarity with the testing stimuli (Freedman et al., 2003, 2006). A problem with these and similar studies was their use of morphing techniques that are now known to not produce dimension differentiation according to psychophysical tests (Folstein et al., 2012). Without such perceptual effects, there is little reason to expect the concomitant changes in neural encoding. In a human neuroimaging study, Folstein et al. (2013) showed that, when the correct morphing procedures are used, not only are changes in early visual cortex observed, but they are found during a task that is different from the trained categorization task (but see Liu & Jagadeesh, 2008, for a case in which such transfer was not observed).

While neuroimaging studies such as that of Folstein et al. (2013) are in line with the hypothesis presented in Figure 9, they do not provide strong support for such a model over alternatives. More research will be necessary to understand the neurocomputational mechanisms underlying dimension differentiation and whether the dimension enhancement hypothesis is in line with them.

### What about changes in readout?

A popular hypothesis regarding the mechanisms of perceptual learning is that, rather than involving changes in the neural populations encoding the relevant stimuli, they involve changes in the way neural populations in later stages of decision processing use the information from the visual encoding populations, what is usually called “readout” mechanisms of learning (e.g., Kahnt et al., 2011; Law & Gold, 2010; Petrov et al., 2005). For example, the model presented in Figure 9 would make the exact same predictions if no changes are assumed in the neural channels encoding the parent faces, but their output was scaled by the readout mechanisms in charge of producing a behavioral decision. Thus, one question that remains to be answered is whether the changes observed in dimension differentiation can also be explained as the result of changes in readout, rather than as changes in existing codes as we have hypothesized here.

There are two reasons to believe that the perceptual changes observed after categorization training with morphed faces and other objects are not due to readout mechanisms. First, the effects of categorization are not task specific, but they transfer from a categorization task to unrelated tasks, both in psychophysical (e.g., Goldstone & Steyvers, 2001; Folstein et al., 2012; Soto & Ashby, 2015; Soto et al., 2017) and neuroimaging (Brants et al., 2016; Folstein et al., 2013) studies. Readout is usually considered task specific, as different tasks are solved optimally using different sources of information. The second reason is that neuroimaging studies have shown that one of the effects of object categorization training is a change in representations located in high-level visual cortex (e.g., Brants et al., 2016; Folstein et al., 2013). Thus, the *enhancement hypothesis* presented in Figure 9 seems more in line with the body of results than a readout hypothesis, although more research directly comparing the predictions of both hypotheses is necessary.

### Differences with encoding of natural face categories

Natural facial categories such as gender, ethnicity, and expression seem to be encoded independently from identity, involving distinct neural populations and their own category-specific norms. Such natural categories also show adaptation effects (Benton et al., 2007; Hsu & Young, 2004; Rutherford et al., 2008; Webster et al., 2004). That is, adaptation to one category value (e.g., male, Caucasian, or disgusted) changes recognition of ambiguous faces away from the adaptor (e.g., ambiguous faces look more female, Asian, or surprised). Those adaptation effects survive changes in identity between the adaptor and the probe stimuli, suggesting that they are not simply due to identity adaptation effects (e.g., Webster et al., 2004; Fox & Barton, 2007).

Several natural face categories seem to be encoded via norm-based opponent channels. For example, adaptation to antiexpressions (created by using an average expression) biases perception toward its corresponding expression, with the effect being stronger when the antiexpressions are farther away from the average, as expected from norm-based coding (Rhodes et al., 2017; Skinner & Benton, 2010). Also in line with norm-based coding, expression aftereffects are specifically in the direction away from the adaptor and toward the average expression (Cook et al., 2011). There is also some evidence that gender encoding is norm based, as the gender adaptation aftereffect is stronger when the adaptor is farther away from the average (Pond et al., 2013).

Thus, on the one hand, natural categories seem to possess their own category-specific codes and norms, independent from identity codes and norms. On the other hand, categorization training does change the perceptual representation of the face identities involved, but our results suggest that it does so by modifying the already existing identity code, rather than creating new codes and norms. Why would natural face categories and artificial face categories behave differently? An important point is that natural categories involve much larger variation in identity than what can be obtained using morphed face dimensions (Soto, 2019). That is, natural categories involve very large perceptual differences between categories and also large perceptual differences within each category. On the other hand, as it is evident from the stimuli shown in Figure 1, morphed face dimensions provide a rather compact perceptual space, in which between-category and within-category variability is small and initially hard to perceive.

A challenge for future work is to develop artificial categorization tasks that involve completely novel face dimensions but also reproduce the larger variability observed in natural categories. For this, one exciting possibility is to move from the use of morphing to the explicit manipulation of three-dimensional face models within a parameterized face space (Hays et al., 2019). This would not only ensure that the tasks are more natural but also facilitate experimental design and data collection. The categorization task used here is extremely difficult to master (this is easy to see from Figure 1), and even with very extensive training, a number of participants are excluded due to low performance. Similarly, the use of morphing to create the cross-adaptation test stimuli in Experiment 2b produced a trade-off between the inclusion of information about the relevant and irrelevant dimensions. This is very different from prior examples of cross-adaptation designs in the literature, in which the relevant and irrelevant dimensions are truly orthogonal (Ellamil et al., 2008; Fox & Barton, 2007; Fox et al., 2008; Webster et al., 2004). The explicit manipulation of three-dimensional face models would allow us to solve these and similar methodological issues.

### Conclusion

In summary, here we used face adaptation aftereffects to explore how categorization training changes the encoding of face identities at the extremes of the category-relevant dimension. Across experiments, the pattern of results suggests that categorization training produces enhancement of already existing dimensions in face space, rather than creation of new category-relevant dimensions. We formalized this hypothesis in a model that explains the most important results in our experiments (see Figure 9) and serves as a working hypothesis for future work in this area. The data and model presented here represent a departure from the most popular interpretation of results from dimension differentiation studies, and we hope that it will motivate new experimental and theoretical research aimed at better understanding the neurocomputational mechanisms behind perceptual changes produced by categorization training.

## Appendix

The model used to create Figure 9 assumes that the two parents of the category-relevant dimension and their anti-faces are each encoded along a dimension that crosses the average in face space, which is a common assumption in studies of encoding of face identity. Each face/anti-face pair is encoded through two channels with opponent stimulus preference, each responding maximally to one of the faces, as shown by the solid curves in Figure 9b. The response of each neural channel as a function or morph level is characterized by a logistic function:

(A1) $$f_{c}\left(s\right)=a_{c}^{max}\frac{1}{1+\exp\left(q_{c}\left(s_{c}-s\right)\right)},$$

where $a_{c}^{max}$ represents the maximum neural activity for channel c, $s_{c}$ is a position parameter that represents the value of expression intensity where the curve is midway between zero and $a_{c}^{max}$, and $q_{c}$ is a parameter determining the slope of the logistic curve. There were a total of four channels c, one for each face and anti-face presented in Figure 2.

The untrained model (solid curves in Figure 9b, top) had parameters $a_{c}^{max}=20$, $s_{c}=0$, and $q_{c}=3$. The trained model (solid curves in 9b, bottom) had the same parameters, with the exception that the channels whose preferred stimuli were the two parents of the category-relevant dimension (c=1,2) had an increased $a_{c}^{max}=40$, which produced an overall scaling up of the channels’ responses.

Adaptation was simply modeled as a scaling down of the responses of the channel whose preferred stimulus was the adaptor (see dotted curves in Figure 9b). The adapted $a_{c}^{max}$ was half the value of the nonadapted $a_{c}^{max}$.

The participant's task in Experiments 2a and 2b was to identify whether a presented face was Parent A or B of the category-relevant dimension or their anti-faces, each with a different name. Because information about each identity in this model is assumed to be dependent on two channels (the identity channel and its anti-identity channel), we first computed decision variables $d_{i}=\frac{f_{i}\left(s\right)}{f_{h}\left(s\right)}$, where i and h represent the channel and anti-channel encoding a given identity i. From these decision variables, we computed the probability of each response $r_{i}$ given the presentation of stimulus s using a simple ratio rule (Luce, 1959):

(A2) $$P\left(r_{i}|s\right)=\frac{d_{i}\left(s\right)}{\sum_{j=1}^{4}d_{j}\left(s\right)}.$$

Adaptation scores were computed from these response probabilities in the same way in which they were computed from response proportions in the data analysis of Experiment 2.
